# Eating patterns of Australian adults: associations with blood pressure and hypertension prevalence

**DOI:** 10.1007/s00394-018-1741-y

**Published:** 2018-06-06

**Authors:** Rebecca M. Leech, Anna Timperio, Anthony Worsley, Sarah A. McNaughton

**Affiliations:** Institute for Physical Activity and Nutrition (IPAN), School of Exercise and Nutrition Sciences, Deakin University, 75 Pigdons Rd, Waurn Ponds, Geelong, 3216 Australia

**Keywords:** Blood pressure, Circadian rhythms, Eating frequency, Eating patterns, Meals, Meal timing, Snacks

## Abstract

**Purpose:**

Eating patterns have been linked to obesity, an established risk factor for hypertension; however, their contribution to hypertension is poorly understood. This study aimed to examine associations of frequency of meals, snacks and all eating occasions (EO), and temporal eating patterns, with blood pressure (BP) and hypertension.

**Methods:**

Dietary data collected via two 24-h recalls during the 2011–2012 Australian National Nutrition and Physical Activity Survey (*n* = 4482 adults, ≥ 19 years) were analysed. Frequencies of EO, meals, and snacks were calculated. Temporal eating patterns were determined using latent class analysis. Multivariate regression models assessed associations of eating patterns with systolic BP (SBP), diastolic BP (DBP), and hypertension prevalence.

**Results:**

Among men, a higher snack frequency was inversely associated with DBP [*β* = − 0.59, 95% confidence interval (CI) (− 1.12, − 0.07)] and hypertension [odds ratio (OR) 0.86, 95% CI (0.75, 0.98)] after adjustment for covariates and BMI. However, these associations disappeared after additional adjustment for total energy intake and overall diet quality. Among women, a temporal eating pattern characterized by a later “lunch” meal was associated with SBP [*β* = 2.45, 95% CI (0.05, 4.84)], DBP [*β* = 1.69, 95% CI (0.25, 3.13)], and hypertension [OR = 1.49, 95% CI (1.00, 2.22)], when compared to a “conventional” eating pattern.

**Conclusions:**

In this study, an inverse association found between snack frequency and BP among men disappeared after adjustment for dietary factors and a “later lunch” pattern was associated with higher BP in women. Future research is needed to understand the relationship and potential mechanistic pathways between eating patterns and BP.

**Electronic supplementary material:**

The online version of this article (10.1007/s00394-018-1741-y) contains supplementary material, which is available to authorized users.

## Introduction

Poor dietary habits, obesity, and high systolic blood pressure (SBP) are three major risk factors contributing to the global burden of disease [1]. As these risk factors are strongly interrelated, it has been postulated that diet is a key driver of global increases in BMI and subsequent SBP [1]. The adoption of a dietary pattern rich in plant-based foods but low in red meat and discretionary (e.g., non-core) foods has been identified as an important strategy for the prevention of cardiovascular disease and hypertension [2, 3]. However, the contribution of eating patterns to hypertension is poorly understood [4]. Eating patterns, including the frequency and temporality of eating occasions [(EO), e.g., meals and snacks], may represent a unique cardiometabolic risk factor [5]. This is because the metabolic and physiological functions in responses to food are also, in part, regulated by circadian clock systems. Thus, variations in the temporal distribution of food consumption may have important health implications [6].

Epidemiologic evidence [7–11] suggests that skipping breakfast and irregular meal habits are associated with poorer cardiometabolic health in adults, including incident hypertension [7]. However, evidence for associations with EO frequency is less consistent [4]. In three studies, a higher EO frequency (e.g., ≥ 5 EO per day) was associated with lower SBP [12, 13] and diastolic blood pressure (DBP) [13, 14]. In support of these findings, a review of short-term experimental studies found that six or more meals per day, compared to one meal, resulted in favourable decreases in cardiometabolic risk markers [15]. However, most of the reviewed studies did not control for differences in dietary intake and other population-based studies have found no evidence for a relation of EO frequency with blood pressure (BP) [16, 17]. Furthermore, research suggests that meals and snacks are differentially related to dietary intakes [18, 19] and obesity [19, 20], yet studies examining meals and snacks as separate EO in relation to hypertension are rare [12].

The temporal distribution of EO in relation to BP has rarely been examined [21, 22]. In the UK Cohort Study, Almoosawi et al. [21] found a positive association for higher total energy intake in the evening with 10-year increases in SBP and DBP. In a study of Spanish adult volunteers, consumption of an afternoon meal was associated with lower SBP and DBP [22]. However, these studies focussed on timing of EO/energy intake at isolated periods of the day and did not simultaneously capture EO at other times of the day [4]. Data-driven statistical techniques (e.g., cluster and latent class analysis) have recently been identified as useful tools for capturing temporal eating patterns across the day, but have only been used to examine relations with diet quality and obesity [23, 24]. Therefore, the aim of this study was to examine the associations of frequency of meals, snacks and all EO, and temporal eating patterns, determined using a latent class analysis approach, with BP and hypertension, in Australian men and women. Based on the findings of our earlier studies that found a positive relation for snacking frequency and a “grazing” temporal eating pattern with measures of adiposity, we hypothesised that that these same eating patterns would be related to (obesity-associated) BP and hypertension [20, 23].

## Subjects and methods

### Sample and study design

**Fig. 1:**
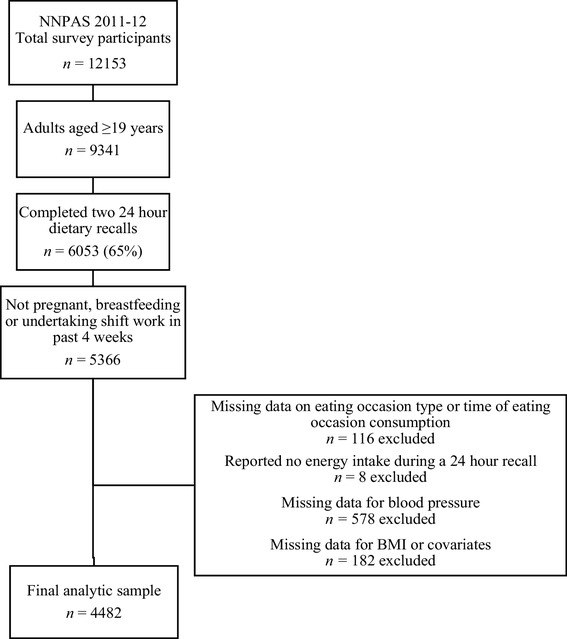
Flowchart of included participants from the 2011–2012 Australian National Nutrition and Physical Activity Survey (NNPAS 2011–12)

This study is a secondary analysis of data drawn from the nationally representative, cross-sectional 2011–2012 Australian National Nutrition and Physical Activity Survey (NNPAS 2011–12) and was registered at anzctr.org.au as ACTRN12617001029381. The NNPAS, conducted between May 2011 and June 2012, was administered by the Australian Bureau of Statistics (ABS); full details of the study design and methods have been described elsewhere [25]. Briefly, 12,153 participants aged 2 or more years (including 9338 adults, aged ≥ 19 years; 77% response rate) were selected using a multistage, probability sampling design of private dwellings (Fig. 1). The Australian Government Census and Statistics Act 1905 provided the ABS with ethics approval to conduct the survey components of the NNPAS [25].

### Blood pressure

All BP measurements were voluntary, excluded pregnant women, and were taken by trained ABS staff during the home visit. Participants were asked to sit comfortably and relax their left arm. Two SBP and DBP measurements were taken on the left arm, with the palm facing upwards, using an automated BP monitor. The second reading was used, except where there was greater than 10 mmHg difference between the first and second readings, in which case, a third reading was taken and the second and third readings averaged. If all three readings differed from each other by > 20 mmHg, then these readings were considered invalid. Participants were classified as hypertensive (≥ 140/90 mmHg) or non-hypertensive (< 140/90 mmHg) [26]. No data about use of BP medications were collected; however, participants’ self-reported whether they had current or previous hypertensive disease.

### Dietary assessment

Dietary data were collected over two 24-h recalls, conducted approximately 9 days apart, on a different day of the week. During each dietary recall, based on the validated USDA automated multiple 5-pass method, participants were asked to identify the type of EO (e.g., breakfast, lunch, dinner, or snack) and the time when each EO commenced. The Australian Supplement and Nutrient Database 2011–2013 was used to determine energy and nutrient intakes from all foods and beverages and dietary information was averaged across the two recall days to obtain mean estimates of eating patterns, total energy intakes, and food intakes.

### Frequencies of all EO, meals, and snacks

The methods used to calculate mean total frequencies of all EO, meals, and snacks have been described previously [18, 27]. Briefly, an EO constituted any eating event that provided a minimum energy content of 210 kJ (50 kcal) and was separated in time from the surrounding EO by 15 min. This approach was informed by the previous research and current recommendations for defining EO in eating patterns research [4, 27]. EO were then classified as meals and snacks according to participants’ self-report of EO. Meals included EO reported as breakfast, brunch, lunch, dinner, and supper, whereas snacks included EO reported as morning/afternoon tea and beverage break were classified as snack EO. Based on the sample distribution, frequencies of all EO, meals and snacks were divided into categories of 1–3, 4–5, or ≥ 6 EO, 1–2, 3, or > 3 meals, and 0–1, 2–3, or > 3 snacks.

### Temporal eating patterns

Temporal eating patterns were determined using latent class analysis, as reported in detail previously [23, 28]. Briefly, three latent classes of temporal eating patterns were identified for men and women based on frequency and timing of EO across the day and labelled according to their defining characteristics. The optimal number of classes was selected based on model fit indices, likelihood ratio tests comparing *k* with *k*^−1^ class models, and pattern interpretability [29]. The first pattern was labelled as “conventional” due to the probability of participants having three EO at “conventional” mealtimes in Australia (e.g., between 7 and 8 a.m., 12–1 p.m., and 6–7 p.m.). The second pattern was distinguished by a > 0.9 probability of a “lunch” meal approximately 1 h later than the “conventional pattern” and labelled the “later lunch” pattern. The probability of a “dinner” meal 1 h later than the conventional pattern was also higher (e.g., > 0.6) in participants with a “later lunch” pattern. The third pattern was labelled the “Grazing” pattern, because it was characterized by frequent but less distinct meal occasions and a higher probability of having an EO after 8 p.m. These temporal eating patterns have been associated with socio-demographic and eating pattern characteristics [28], diet quality, and, among women, adiposity outcomes [23].

### Covariates

The following socio-demographic, health behaviours, and anthropometric variables, collected during the household survey, were considered as potential covariates due to their previous reported relation with hypertension risk [1, 26].

#### Socio-demographics

Education level was categorised as: low (completed some high-school or less), medium (completed high-school or completed some high-school and/or certificate/diploma) or high (having a tertiary qualification). Country of birth was categorised by the ABS as: Australia, predominantly English-speaking countries (other than Australia) and all other countries.

#### Health behaviours

Smoking status was self-reported and categorised as current smoker, ex-smoker, and never smoked. Participants were categorised as meeting or not meeting current Australian physical activity guidelines (150 min and 5 sessions), based on self-reported frequency and duration of walking for recreation or transport and moderate or vigorous leisure-time physical activity [30, 31]. Self-reported information on sleep duration the night before the survey and how much time participants spent sitting or lying down at work, during transport and leisure activities in the past week, were used to calculate (per day) total minutes spent sleeping per day and in sedentary behaviour, respectively. Participants reported whether they were currently on a weight-loss diet for health reasons (yes/no). Average daily total energy intake and diet quality scores were calculated from the 2 days of recall. The established food-based Dietary Guidelines Index (DGI) was used as a measure of overall diet quality [32–34]. The DGI assesses compliance with recommendations outlined in the Australian Dietary Guidelines, and is the sum of 13 components (score range of 0–130), each corresponding to an Australian Dietary guideline and scored proportionally out of 10. The components include meeting recommendations for food variety, intakes of fruits, vegetables (including legumes), grain foods, dairy and alternatives, meat and alternatives, unsaturated fat, fluids, discretionary foods, saturated fat, salt, added sugar, and alcohol. Higher scores indicate a better diet quality. Measurement of height (cm) and weight (kg) were taken to one decimal point by trained ABS staff using a portable stadiometer and digital scales. BMI (weight [kg]/height [m]^2^) was calculated.

### Analytic sample

The analytic sample included the 65% of adult participants who completed both dietary recalls (*n* = 6053; Fig. 1). Participants were eligible for this analysis if they were not pregnant, breastfeeding, or undertaking shift-work in the past 4 weeks (*n* = 5366) and were excluded if they reported no energy intake during either dietary recall (*n* = 8 excluded) or did not report the time at which an EO commenced or the type of EO (*n* = 116 excluded). Of the remaining 5242 participants, 578 (11%) had missing data for BP and a further 182 (3.9%) were missing data for BMI and covariates: BMI (*n* = 149), physical activity (*n* = 28), and sedentary time (*n* = 5). The final analytic sample was 2099 men and 2383 women.

### Statistics

All statistical analyses were stratified by sex and used Stata statistical software, Version 14.2 (Stata Inc., College Station, TX, USA). Point estimates and standard errors were determined by applying person and replicate weights that accounted for the probability of participant selection and the clustered survey design, respectively. Descriptive statistics for sample characteristics are presented as weighted means (95% CI) or weighted percentages. After examining the distribution of the data, the following variables were log-transformed to improve normality: BMI, daily total sedentary time, and total energy intake. Weighted geometric means (95% CI) were used for all log-transformed variables.

The *F* test (for continuous data) and adjusted Pearson *χ*^2^ test (for categorical data) were used to determine sex-specific differences in sample characteristics by hypertension status. Multiple linear regression (for continuous outcomes) and logistic regression (for binary outcomes) were used to test for associations of frequencies of all EO, meal and snacks (continuous), and temporal eating patterns, with SBP and DBP (continuous) and hypertension prevalence (binary). Four models were tested: model 1 was an unadjusted model; model 2 adjusted for age (years, continuous), education level (low, medium or high), country of birth (Australia, other predominantly English-speaking countries, all other countries), smoking status (never, former or current), daily, meeting physical activity guidelines (yes/no), daily sedentary time (min; continuous), sleep duration (h, continuous), dieting for health reasons (yes/no); model 3 further adjusted for BMI, and model 4 further adjusted for total energy intake and DGI scores (both continuous). In light of the previous research that reported a positive association among participants with the highest EO frequencies (i.e., > 5 EO) [13], in the present study, any observed statistically significant (*P* < 0.05) adjusted association between the continuous measures of frequencies of EO, meals, or snacks, and the outcome variables were further explored by examining associations for eating pattern frequency categories (e.g., 1–3 [reference], 4–5 or ≥ 6 EO; 1–2 [reference], 3 or > 3 meals and 0–1 [reference], and 2–3 or > 3 snacks). Finally, the effect of energy misreporting, defined as the ratio of total energy intake to total energy expenditure was considered [35]; however, its inclusion did not improve its predictive power when BMI was already in the model. A previous study has also shown that energy misreporting bias can be statistically corrected using predictors of energy misreporting (i.e., dieting behaviours and BMI) [36].

### Sensitivity analysis

As data on BP medications use were not collected in the NNPAS [25], a sensitivity analysis was conducted that included only participants who self-reported no current or previous hypertensive disease (*n* = 1612 men and *n* = 1853 women).

## Results

Table 1 presents the characteristics of men and women participants in the NNPAS 2011–12 by hypertension status. Of the participants, 24% of men and 20% of women were classified as having hypertension. Among both sexes, there were significant differences by hypertension status for age, education level, meeting physical activity guidelines, BMI, and self-report status of previous or current hypertensive disease (*P* < 0.05). Differences were also found for total daily energy intake, DGI scores, and frequency of all EO and snacks between non-hypertensive and hypertensive men (*P* < 0.05). For the snack frequency categories, a higher and lower proportion of hypertensive men reported having fewer than two snacks and more than three snacks per day, respectively, compared to non-hypertensives (*P* < 0.05). No significant differences were found among women for the dietary or eating pattern variables according to hypertension status.

**Table 1:** Characteristics of men and women in the NNPAS by hypertension status

	Men (*n* = 2099)			Women (*n* = 2383)		
	Non-hypertensive (*n* = 1538)	Hypertensive (*n* = 561)	*P* value	Non-hypertensive (*n* = 1883)	Hypertensive (*n* = 500)	*P* value
Socio-demographics						
Age (years)	43.1 (42.4, 44.8)	56.2 (54.2, 58.2)	< 0.0001	44.7 (44.0, 45.5)	58.4 (56.8, 60.0)	< 0.0001
Education level (%)			< 0.05			< 0.0001
Low	18	25		25	43	
Medium	53	54		44	33	
High	29	21		32	24	
Country of birth (%)			0.95			0.12
Australia	69	69		70	61	
Predominantly English-speaking countries	13	13		11	15	
All other countries	18	18		19	24	
Health behaviours or characteristics						
Smoking status			0.14			0.56
Never	47	39		59	55	
Former	35	40		27	31	
Current	18	20		14	14	
Meets physical activity guidelines^a^ (%)	48	41	0.04	45	35	< 0.01
Daily sedentary time^b^ (min)	306.7 (289.8, 324.6)	297.2 (273.0, 323.4)	0.54	260.3 (247.9, 273.2)	263.1 (237.0, 292.1)	0.86
Currently on a diet for health reasons (%)	12	8	0.06	17	15	0.43
Sleep duration (h)	7.9 (7.8, 8.0)	7.9 (7.7, 8.0)	0.81	8.0 (7.9, 8.1)	8.0 (7.7, 8.2)	0.52
Total energy intake^b^ (kJ)	9498 (9304, 9696)	8463 (8170, 8766)	< 0.0001	7087 (6928, 7250)	6890 (6587, 7206)	0.31
Dietary Guidelines Index^c^ (score)	80.1 (79.0, 81.2)	77.6 (75.7, 79.6)	< 0.05	80.7 (79.5, 82.0)	81.8 (80.1, 83.5)	0.31
BMI^b^ (score)	26.8 (26.5, 27.2)	28.9 (28.2, 29.5)	< 0.0001	25.9 (25.5, 26.3)	28.7 (27.9, 29.6)	< 0.0001
Systolic blood pressure (mmHg)	118.7 (117.9, 119.5)	148.3 (145.6, 151.0)	< 0.0001	112.5 (111.7, 113.1)	148.8 (146.9, 150.8)	< 0.0001
Diastolic blood pressure (mmHg)	73.4 (72.8, 74.0)	88.7 (87.5, 89.8)	< 0.0001	72.9 (72.2, 73.6)	89.0 (87.9, 90.2)	< 0.0001
No current or previous hypertensive disease (%)	85	63	< 0.0001	86	56	< 0.0001
Eating patterns						
Eating occasion frequency	4.9 (4.8, 5.0)	4.7 (4.5, 4.8)	< 0.01	4.8 (4.7, 4.9)	4.7 (4.6, 4.9)	0.37
Meal frequency	2.9 (2.8, 2.9)	2.9 (2.8, 2.9)	0.79	2.9 (2.9, 3.0)	3.0 (2.9, 3.0)	0.55
Snack frequency	2.1 (1.98, 2.2)	1.8 (1.7, 2.0)	< 0.01	1.9 (1.9, 2.0)	1.8 (1.7, 2.0)	0.19
Categories of eating occasion frequency (%)			0.07			0.54
1–3	19	24		18	20	
4–5	57	60		61	62	
≥ 6	23	17		21	18	
Categories of meal frequency (%)			0.27			0.89
< 3	31	32		26	25	
3	53	48		54	54	
> 3	15	20		20	21	
Categories of snack frequency (%)			< 0.05			0.59
< 2	45	51		48	51	
2–3	37	38		39	38	
> 3	18	11		13	11	
Latent classes of temporal eating patterns (%)			0.09			0.17
Conventional	40	47		41	37	
Later lunch	36	30		32	39	
Grazing	24	23		27	24	

Values are weighted means (95% confidence intervals) or weighted percentages. Significant sex-specific differences by hypertension status assessed using an *F* test for continuous variables or design-adjusted Pearson *χ*^2^ test

^a^Whether met physical activity guidelines of 150 min and 5 sessions/week

^b^Values are geometric means (95% CI)

^c^DGI represents a total diet quality score (score range 0–130) with higher scores indicating better overall diet quality

The sex-specific associations of frequency of all EO, meals, and snacks (continuous) with SBP and DBP are presented in Table 2. In the basic model, a statistically significant positive association was found between meal frequency and SBP among women which disappeared after adjustment for the covariates in model 2. Among men, inverse associations were found between frequency of all EO and snacks and DBP, and after adjustment for covariates (model 2) and BMI (model 3).

**Table 2:** Associations of eating patterns with systolic and diastolic blood pressure in Australian men and women

	Systolic blood pressure (mmHg)				Diastolic blood pressure (mmHg)			
	Model 1^a^	Model 2^b^	Model 3^c^	Model 4^d^	Model 1^a^	Model 2^b^	Model 3^c^	Model 4^d^
Men (*n* = 2099)								
Eating frequency								
Eating occasion frequency	− 0.49 (− 1.22, 0.25)	− 0.65 (− 1.41, 0.11)	− 0.47 (− 1.24, 0.30)	− 0.18 (− 1.11, 0.74)	− 0.80 (− 1.36, − 0.23)**	− 0.88 (− 1.45, − 0.31)**	− 0.63 (− 1.17, − 0.09)*	− 0.39 (− 0.98, 0.20)
Meal frequency	0.47 (− 1.96, 2.91)	− 1.29 (− 3.41, 0.84)	− 0.86 (− 3.13, 1.40)	0.13 (− 2.35, 2.62)	− 1.04 (− 2.43, 0.35)	− 1.46 (− 2.99, 0.08)	− 0.88 (− 2.48, 0.71)	− 0.46 (− 2.18, 1.26)
Snack frequency	− 0.69 (− 1.46, 0.09)	− 0.59 (− 1.37, 0.19)	− 0.46 (− 1.22, 0.30)	− 0.32 (− 1.24, 0.59)	− 0.73 (− 1.33, − 0.13)*	− 0.77 (− 1.35, − 0.20)**	− 0.59 (− 1.12, − 0.07)*	− 0.41 (− 0.99, 0.18)
Temporal eating patterns								
Conventional (reference, *n* = 941)	–	–	–	–	–	–	–	–
Later lunch (*n* = 702)	− 2.44 (− 4.93, 0.05)	− 1.13 (− 3.42, 1.16)	− 0.80 (− 2.94, 1.33)	− 0.68 (− 2.82, 1.46)	− 0.97 (− 2.66, 0.71)	− 0.63 (− 2.37, 1.11)	− 0.19 (− 1.74, 1.36)	− 0.10 (− 1.65, 1.45)
Grazing (*n* = 456)	− 2.72 (− 5.59, 0.15)	0.63 (− 2.19, 3.46)	0.77 (− 1.89, 3.42)	0.75 (− 1.86, 3.36)	− 1.61 (− 3.86, 0.64)	− 0.99 (− 3.19, 1.21)	− 0.81 (− 2.68, 1.05)	− 0.58 (− 2.49, 1.34)
Women (*n* = 2383)								
Eating frequency								
Eating occasion frequency	− 0.07 (− 1.06, 0.91)	− 0.07 (− 0.88, 0.75)	− 0.06 (− 0.75, 0.86)	− 0.51 (− 1.46, 0.43)	− 0.02 (− 0.58, 0.54)	− 0.03 (− 0.58, 0.52)	− 0.10 (− 0.47, 0.67)	− 0.27 (− 0.88, 0.35)
Meal frequency	2.33 (0.06, 4.59)*	− 0.13 (− 2.03, 1.78)	0.14 (− 1.82, 2.09)	− 0.76 (− 2.86, 1.33)	− 0.07 (− 1.72, 1.58)	− 0.28 (− 1.83, 1.27)	− 0.01 (− 1.55, 1.53)	− 0.65 (− 2.15, 0.85)
Snack frequency	− 0.62 (− 1.73, 0.50)	− 0.08 (− 1.01, 0.84)	0.00 (− 0.92, 0.92)	− 0.52 (− 1.56, 0.52)	0.03 (− 0.54, 0.61)	0.07 (− 0.51, 0.64)	0.15 (− 0.43, 0.74)	− 0.16 (− 0.81, 0.49)
Temporal eating patterns								
Conventional (reference, *n* = 1001)	–	–	–	–	–	–	–	–
Later lunch (*n* = 807)	2.13 (− 0.72, 4.97)	2.38 (− 0.01, 4.77)	2.55 (0.12, 4.97)*	2.45 (0.05, 4.84)*	1.69 (0.16, 3.21)*	1.56 (0.11, 3.01)*	1.73 (0.27, 3.19)*	1.69 (0.25, 3.13)*
Grazing (*n* = 575)	0.08 (− 2.76, 2.93)	2.41 (− 0.27, 5.10)	2.20 (− 0.52, 4.92)	1.93 (− 0.87, 4.72)	0.98 (− 0.69, 2.66)	0.85 (− 0.83, 2.54)	0.64 (− 1.08, 2.35)	0.50 (− 1.22, 2.22)

Values are presented as *β* coefficients (95% confidence intervals). Associations were examined using the Wald tests of associations for linear regression; *P < 0.05, **P < 0.01

^a^Crude analysis

^b^Adjusted for age (years, continuous), sedentary time (min/day, continuous), education level (low/medium/high), country of birth (Australia/other mainly English-speaking countries/all other countries), meets PA guidelines (yes/no), smoking status (never smoked/past smoker/current smoker), and dieting (yes/no)

^c^Model 2 and additionally adjusted for BMI scores

^d^Model 3 and additionally adjusted for Dietary Guideline Index scores and total energy intake

After further analysis that examined these associations by categories of snack and EO frequency, the inverse associations were observed among men who reported > 3 snacks [DBP: *β* = − 2.23, 95% CI (− 4.14, − 0.32); *P* = 0.023] and ≥ 6 EO [DBP: *β* = − 2.32, 95% CI (− 4.45, − 0.20); *P* = 0.032], compared to those who reporting < 2 snacks and ≤ 3 EO. However, these associations with DBP for men were attenuated after further adjustment for the total energy intake and DGI scores: *β* = − 1.56, 95% CI (− 3.59, 0.47); *P* = 0.13 and *β* = − 1.37, 95% CI (− 3.62, 0.89); *P* = 0.23, respectively.

Results of the regression analyses showed no associations between latent classes of temporal eating patterns and SBP or DBP among men (Table 2). Among women, a “later lunch” temporal eating pattern was positively associated with SBP and DBP, when compared to a “conventional” pattern, after adjustment for covariates and BMI, and after further adjustment for dietary intakes.

Associations of frequency of all EO, meals, and snacks (continuous) and latent classes of temporal eating patterns with hypertension prevalence are shown in Table 3. Among men, a significant inverse association was found for frequency of all EO and snacks but not meals.

**Table 3:** Associations of eating patterns with hypertension prevalence in Australian men and women

	Model 1^a^	Model 2^b^	Model 3^c^	Model 4^d^
Men (*n* = 2099)				
Eating frequency				
Eating occasion frequency	0.87 (0.80, 0.97)*	0.85 (0.75, 0.96)**	0.87 (0.76, 0.98)*	0.94 (0.81, 1.08)
Meal frequency	1.03 (0.76, 1.40)	0.88 (0.64, 1.21)	0.92 (0.66, 1.30)	1.12 (0.77, 1.61)
Snack frequency	0.86 (0.76, 0.96)*	0.85 (0.75, 0.96)*	0.86 (0.75, 0.98)*	0.91 (0.79, 1.05)
Temporal eating patterns				
Conventional (reference, *n* = 941)	–	–	–	–
Later lunch (*n* = 702)	0.70 (0.52, 0.93)*	0.78 (0.56, 1.08)	0.81 (0.59, 1.12)	0.82 (0.59, 1.13)
Grazing (*n* = 456)	0.81 (0.55, 1.20)	1.14 (0.74, 1.74)	1.15 (0.76, 1.75)	1.22 (0.81, 1.82)
Women (*n* = 2383)				
Eating frequency				
Eating occasion frequency	0.94 (0.83, 1.08)	0.95 (0.83, 1.08)	0.97 (0.84, 1.10)	0.93 (0.79, 1.09)
Meal frequency	1.10 (0.81, 1.50)	0.94 (0.70, 1.27)	0.96 (0.70, 1.31)	0.93 (0.79, 1.09)
Snack frequency	0.91 (0.79, 1.05)	0.95 (0.83, 1.09)	0.95 (0.82, 1.10)	0.93 (0.79, 1.09)
Temporal eating patterns				
Conventional (reference, *n* = 1001)	–	–	–	–
Later lunch (*n* = 807)	1.34 (0.94, 1.90)	1.47 (1.00, 2.16)	1.51 (1.01, 2.25)*	1.49 (1.00, 2.22)*
Grazing (*n* = 575)	0.97 (0.64, 1.45)	1.11 (0.71, 1.73)	1.06 (0.67, 1.68)	1.04 (0.64, 1.67)

Values are presented as odds ratios (95% confidence intervals). Associations were examined using the Wald tests of associations for logistic regression; *P < 0.05, **P < 0.01

^a^Crude analysis

^b^Adjusted for age (years, continuous), sedentary time (min/day, continuous), education level (low/medium/high), country of birth (Australia/other mainly English-speaking countries/all other countries), meets PA guidelines (yes/no), smoking status (never smoked/past smoker/current smoker), and dieting (yes/no)

^c^Model 2 and additionally adjusted for BMI scores

^d^Model 3 and additionally adjusted for Dietary Guideline Index scores and total energy intake

After further analysis that examined these associations by categories of snack and EO frequency, the inverse adjusted associations (model 3) were only observed among men who reported > 3 snacks [OR 0.52, 95% CI (0.30, 0.81); *P* = 0.006] and ≥ 6 EO [OR 0.54, 95% CI (0.34, 0.84); *P* = 0.008], compared to those who reporting < 2 snacks and ≤ 3 EO. However, again, these associations attenuated after further adjustment for total energy intake and DGI scores, and no significant associations were found among women. Compared to a “conventional” temporal eating pattern, a “later lunch temporal” eating pattern was also associated with hypertension prevalence among women, but only after adjustment for covariates and BMI scores.

Results of the sensitivity analyses which included only men and women with no self-reported current or previous hypertensive disease, showed similar null associations of frequency of EO, snacks, and meals with SBP, DBP or hypertension prevalence, after adjustment for covariates, BMI, and dietary intakes (Supplementary Table 1). However, among women, the finding of a positive association between a “later lunch” pattern and DBP (but not SBP or hypertension prevalence) persisted after exclusion of those with no self-reported current/previous hypertensive disease.

## Discussion

To our knowledge, this is one of the first studies among adults to examine associations of meal and snack frequency and temporal eating patterns, based on the timing and frequency of EO across the day, with BP and hypertension prevalence [12]. Among men, frequency of all EO and snacks was inversely associated with DBP and lower odds of hypertension prevalence, but these associations disappeared after adjustment for overall diet quality scores and total energy intakes. Among women, a “later lunch” pattern, identified using latent class analysis in our earlier study [28], and characterized by a later lunch EO (e.g., between 1 and 2 p.m.) was associated with higher SBP, DBP, and hypertension prevalence. However, only associations with DBP persisted after exclusion of persons with the self-reported previous/current hypertension.

Only a few studies have examined the relationship between EO frequency and BP among adults [12, 13, 16, 17], with conflicting findings. However, it is difficult to compare the results of these studies, because they define EO using different approaches. For example, EO have mostly been self-reported by participants in response to a single survey question where an EO is not further defined [12, 17]. Whereas in another study, in a small sample of healthy volunteers (*n* = 115), EO was defined as any eating event (including kilojoule-free events) separated in time by 15 min [13]. A higher EO frequency has been associated with lower hypertension prevalence [12] and incidence [13], whereas other studies have found no associations with BP [16, 17]. In the present study, EO frequency was inversely associated with DBP and hypertension prevalence among men, but these associations attenuated after further adjustment for total energy intakes and overall diet quality.

Studies examining the separate effects of meal and snack frequency on BP outcomes are rare [12]. Compared to participants who reported no snacks, Kim et al. [12] found that a snack frequency of three per day was associated with lower odds of hypertension, but associations attenuated (e.g., 95% CI included one) after adjustment for adiposity measures. In the present study, snack frequency (specifically > 3 snacks) was inversely associated with BP outcomes among men, but again associations attenuated after adjustment for overall diet quality and total energy intakes. Notably, with respect to adjustment of dietary factors, the previous studies on eating patterns and BP have only adjusted for either total energy intakes [13] or energy and nutrient intakes [12, 16, 17], and not a measure of overall diet quality based on food intakes.

The findings from the present study suggest that diet quality may be an important factor in the relation between EO frequency and BP, and is supported by the previous studies that have shown a beneficial effect of healthful dietary patterns for the prevention of hypertension [37]. In a previous study of NNPAS participants. [18], snack frequency was associated with better diet quality scores for intakes of fruits and dairy products, two food groups recommended as part of the Dietary Approaches to Stop Hypertension (DASH) diet [3]. However, in the same study, snack frequency was also associated with poorer scores for intakes of discretionary foods and added sugars among men [18]. Future research that examines the role of diet quality on the relation between EO frequency and hypertension is warranted.

Epidemiological evidence suggests a positive association between evening energy intakes or the later timing of an EO and obesity [38], but studies examining temporal patterns of eating in relation to BP are rare. In one study, higher energy intake at breakfast was associated with lower hypertension prevalence but not 10-year incidence, and higher energy intake in the evening was associated with higher hypertension incidence, which remained borderline significant after adjustment for baseline BMI [21]. Another study, in the same cohort, found no association between time of day macronutrient intakes and BP [39]. Keller et al. found that the consumption of an afternoon meal, but not other conventional Spanish meals, was modestly associated with lower SBP and DBP, even after adjustment for dietary intake and waist circumference [22]. In the present study, a temporal eating pattern characterized by having a later “lunch” meal was associated with SBP, DBP, and hypertension prevalence among women, after adjustment for potential covariates, BMI, and diet quality. However, rather than examining the timing of a single meal or energy intake across stratified time-periods, the present study examined temporal eating patterns based on a novel latent class analysis approach, which captures the timing multiple EO across the day and the likely correlations of energy intakes between EO [4].

The possible mechanisms by which the later timing of a “lunch” meal might increase SBP and DBP among women are unclear. The timing of the “dinner” (evening) meal also tended to be later in women with this pattern and research has shown that insulin sensitivity gradually lowers across the day, into the evening [40]. In addition, in an experimental trial that controlled for dietary intakes, the timing of an evening meal high in energy and carbohydrates but low in fibre was associated with reduced insulin sensitivity and higher blood glucose levels, when compared to participants who had the same meal in the morning [41]. Insulin metabolism may contribute to the association between temporal eating patterns and BP due to its role in modulating vasodilator effects on the endothelium via nitric oxide bioavailability [40]. Measures of insulin sensitivity and blood glucose levels in future epidemiological research examining temporal eating patterns and BP are needed.

Strengths of this study include the examination of associations with BP in a large nationally representative sample, adjusted for BMI, and multiple important confounders, including a measure of overall diet quality. Eating patterns were determined from 2 days of dietary recall, and an EO was defined using an evidence-based approach [27]. While the novel methodology used to determine temporal eating patterns is also considered a study strength, it should be noted that findings from data-driven, exploratory methods may not be generalizable to populations from other countries. For example, the temporal eating patterns found in the present study may reflect the sociocultural and lifestyle characteristics of the Australian adult population; further research is needed to better understand differences in temporal eating patterns in other populations. Limitations of the present study include the cross-sectional study design which does not permit the assessment of temporal relationships and the lack of data on anti-hypertensive medications which may have led to an underestimation of associations with BP. However, in the analysis that excluded participants with no current/previous self-reported hypertension, the association between a “later lunch pattern” with DBP persisted.

In conclusion, a temporal eating pattern distinguished by a later “lunch” meal when compared to a “conventional” Australian mealtime pattern was associated with higher BP in women. Among men, a higher snack frequency was inversely associated with BP, but these associations disappeared after adjustment for total energy intake and overall diet quality. Future research that considers the role of overall diet quality and cardiometabolic indicators (e.g. insulin and glucose levels) on the relation between eating patterns and BP is warranted.

## Electronic supplementary material

Below is the link to the electronic supplementary material.

Supplementary material 1 (DOCX 15 KB)
