# Supplementary material for: Eating patterns of Australian adults: associations with blood pressure and hypertension prevalence
Source: Eur J Nutr. 2018 Jun 6;58(5):1899–909. doi: 10.1007/s00394-018-1741-y (PMC6647126; doi:10.1007/s00394-018-1741-y)
Supplement: Supplementary file 1 — Supplementary material 1 (DOCX 15 KB) [file 394_2018_1741_MOESM1_ESM.docx]

**Supplementary Table 1:** Associations of eating patterns with systolic blood pressure, diastolic blood pressure and hypertension prevalence in Australian men and women with no self-reported current or previous hypertensive disease^a^

|  | **Systolic blood pressure (mmHg)** | **Diastolic blood pressure (mmHg)** | **Hypertension** |
| --- | --- | --- | --- |
|  | *β-coefficient (95% CI)* | *β-coefficient (95% CI)* | *Odds Ratio (95% CI)* |
| *Men* ***(n=1612)*** |  |  |  |
| Eating frequency |  |  |  |
| Eating occasion frequency | -0.55 (-1.52, 0.43) | -0.39 (-0.98, 0.20) | 0.86 (0.70, 1.06) |
| Meal frequency | -0.27 (-3.20, 2.66) | -0.85 (-2.77, 1.08) | 1.05 (0.61, 1.81) |
| Snack frequency | -0.67 (-3.20, 2.66) | -0.34 (-0.91, 0.22) | 0.83 (0.67, 1.02) |
| Temporal eating patterns |  |  |  |
| Conventional pattern (reference, n=705) | **-** | - | 1.00 |
| Later Lunch pattern (n=553) | -0.88 (-2.77, 1.01) | 0.24 (-1.30, 1.79) | 0.81 (0.53, 1.25) |
| Grazing pattern (n=354) | 0.41 (-2.29, 3.12) | 0.37 (-1.61, 2.35) | 1.33 (0.82, 2.17) |
| *Women* ***(n=1853)*** |  |  |  |
| Eating frequency |  |  |  |
| Eating occasion frequency | -0.55 (-1.49, 0.38) | -0.28 (-1.01, 0.44) | 0.85 (0.70, 1.04) |
| Meal frequency | -0.26 (-2.47, 1.96) | -0.25 (-1.63, 1.14) | 1.01 (0.65, 1.58) |
| Snack frequency | -0.61 (-2.47, 1.96) | -0.19 (-0.96, 0.57) | 0.83 (0.69, 1.01) |
| Temporal eating patterns |  |  |  |
| Conventional pattern (reference, n=753) | **-** | - | 1.00 |
| Later Lunch pattern (n=626) | 2.08 (-0.41, 4.58) | 1.67 (0.25, 3.09)^*^ | 1.44 (0.85, 2.43) |
| Grazing pattern (n=474) | 0.67 (-2.18, 3.52) | 0.19 (-1.64, 2.01) | 0.66 (0.33, 1.30) |

^a^Associations were examined using Wald tests of associations for linear or logistic regression, adjusted for: age (y, continuous), sedentary time (min/d, continuous), education level (low/medium/high), country of birth (Australia/other mainly English-speaking countries/all other countries), meets PA guidelines (YES/NO), smoking status (never smoked/past smoker/current smoker), dieting (YES/NO), BMI scores, Dietary Guidelines Index scores, total energy intake and

^*^P<0.05
